# Retention in an antiretroviral therapy programme during an era of decreasing drug cost in Limbe, Cameroon

**DOI:** 10.1186/1758-2652-14-32

**Published:** 2011-06-16

**Authors:** Jembia J Mosoko, Wilfred Akam, Paul J Weidle, John T Brooks, Asabi J Aweh, Thompson N Kinge, Sherri Pals, Pratima L Raghunathan

**Affiliations:** 1 Division of Global HIV/AIDS, Centers for Disease Control and Prevention, Mutengene, Cameroon; 2 Limbe Regional Hospital, Ministry of Public Health, Cameroon; 3 Division of HIV/AIDS Prevention, National Center for HIV, Viral Hepatitis, STD, and TB Prevention, Centers for Disease Control and Prevention, Atlanta, Georgia, USA

## Abstract

**Background:**

In 2002, Cameroon initiated scale up of antiretroviral therapy (ART); on 1 October 2004, a substantial reduction in ART cost occurred. We assessed the impact of this event and other factors on enrolment and retention in care among HIV-infected patients initiating ART from February 2002 to December 2005 at the single ART clinic serving the Southwest Region in Limbe, Cameroon.

**Methods:**

We retrospectively analyzed clinical and pharmacy payment records of HIV-infected patients initiating ART according to national guidelines. We compared two cohorts of patients, enrolled before and after 1 October 2004, to determine if price reduction was associated with enhanced enrolment. We assessed factors associated with retention and survival by Cox proportional hazards models. Retention in care implied patients who had contact with the healthcare system as of 31 December 2005 (including those who were transferred to continue care in other ART centres), although these patients may have interrupted therapy at some time. A patient who was not retained in care may have dropped out (lost to follow up) or died.

**Results:**

Mean enrolment rates for 2920 patients who initiated ART before and after the price reduction were 46.5 and 95.5 persons/month, respectively (p < 0.001). The probabilities of remaining alive and in care were 0.66 (95% CI 0.64-0.68) at six months, 0.58 (95% CI 0.56-0.60) at one year, 0.47 (95% CI 0.45-0.49) at two years and 0.35 (95% CI 0.32-0.38) at three years; they were not significantly different between the two cohorts of patients enrolled before and after the price reduction over the first 15 months of comparable follow up (hazard ratio 1.1; 95% CI 0.9-1.2, p = 0.27). In multivariable analysis using multiple imputations to compensate for missing values, factors associated with dropping out of care or dying were male gender (HR 1.33 [1.18-1.50], p = 0.003), treatment paid by self, family or partly by other (HR 3.05 [1.99-4.67], p < 0.001), and, compared with residents of Limbe, living more than 150 km from Limbe (HR 1.41 [1.18-1.69], p < 0.001), or being residents of Douala (HR 1.51 [1.16-1.98], p < 0.001).

**Conclusions:**

Reducing the cost of ART increased enrolment of clients in the programme, but did not change retention in care. In a system where most clients pay for ART, an accessible clinic location may be more important than the cost of medication for retention in care. Decentralizing ART clinics might improve retention and survival among patients on ART.

## Background

Nearly two-thirds of the world's estimated 33 million HIV-infected adults live in sub-Saharan Africa [1]. In response to this situation, the World Health Organization (WHO), in collaboration with developing countries, recommended the "3 by 5" initiative: treatment of 3 million people living with HIV/AIDS by the end of 2005 [2].

Treatment of HIV-infected patients with antiretroviral therapy (ART) has been shown to suppress viral replication and dramatically extend survival [3-9]. Despite its demonstrated benefits, access to ART remains limited in resource-constrained settings. ART programmes in developing countries face many challenges, including large patient burdens, costly drugs, inadequate numbers of trained healthcare staff, and laboratory facilities ill-equipped to monitor patients receiving ART. For the estimated 4 million HIV-infected patients who needed therapy in sub-Saharan Africa in 2006, coverage was 16% [10]. In Cameroon, 46,000 (25%) of the estimated 180,000 HIV-infected patients eligible for ART were receiving treatment in 2007 [11].

Since 2002, Cameroon has undertaken an initiative to scale up access to ART. From 2002 until 2005, most patients paid for ART themselves in Cameroon. On 1 October 2004, the cost to patients of ART was reduced substantially. From 1999 to 2005, the average monthly cost of first-line ART decreased by more than 100-fold, while the per capita gross national income increased from US$590 to US$630 [12] (World Bank 2004). Cameroon benefited from the Accelerating Access Initiative, which reduced the initial price of antiretroviral drugs from US$915 to US$128 per month in 2000. The initiative was launched in May 2000 with six pharmaceutical firms and five United Nations agencies (UNAIDS, the World Bank, WHO, UNICEF and UNFPA).

Following negotiations with pharmaceutical firms and subsidies from the government of Cameroon, the price further decreased to between US$27.40 and US$51.20 per month in 2002. With Cameroon's successful proposal to the Global Fund to Fight AIDS, Tuberculosis and Malaria (Global Fund), in which care for HIV-infected patients was an integral component, on 1 October 2004 the price of antiretroviral drugs declined further to US$5.50 and US$12.80 per month for first-and second-line treatment regimens, respectively. Treatment units were allowed to enrol up to 15% of all treated persons for free ART. Global Fund-supported first-and second-line ART regimens included nucleoside and non-nucleoside reverse transcriptase inhibitor-based and protease inhibitor-based ART.

Before 2005, the Limbe Regional Hospital ART clinic was the sole clinic that provided ART in the Southwest Region of Cameroon, a region that covers 25,410 km^2 ^with an estimated population of 1,100,000 inhabitants [13]. As of December 2005, this ART clinic had enrolled and initiated ART for more than 3000 HIV-infected patients, representing about 17% of patients receiving ART in Cameroon. In order to assess this programme's performance and to identify opportunities for improvement, we analyzed factors associated with enrolment and retention in care among HIV-infected patients initiating ART between February 2002 and December 2005 at this clinic.

## Methods

### Antiretroviral therapy programme enrolment

HIV-infected patients were eligible for enrolment in the Limbe ART programme if they met the following criteria according to national guidelines: AIDS-defining illness or symptomatic HIV disease or CD4 count of more than 200 cells/mm^3^. The clinical staging used for entry into the ART programme was changed from the US Centers for Disease Control and Prevention (CDC) classification to the WHO classification in 2005; therefore, persons were eligible for ART if they had CDC stage A3, B2, B3 or C disease, or WHO clinical stages 3 and 4 disease. ART was dispensed monthly, although for patients for whom transportation to the clinic was difficult, the clinic providers could dispense two to three months of medication.

### Procedures

We analyzed data that had been abstracted from patients' medical charts using standardized forms. These data were entered and maintained in a single analysis dataset (Microsoft Excel 2003, Microsoft Corp, Redmond, Washington, USA) and included age, sex, past medical history, prior treatment history (e.g., ART, opportunistic infection prophylaxis, tuberculosis treatment), functional status and WHO/CDC clinical stage, presenting signs and symptoms, weight, entry and follow-up CD4 cell counts, results of complete blood counts (e.g., haemoglobin, white blood cell count), blood chemistries (e.g., serum creatinine, liver transaminases), and enrolment patterns (i.e., attendance).

To characterize access to the clinic, patients were categorized into those who: 1) were residents of Limbe; 2) lived less than 40 km from Limbe; 3) lived 40-150 km from Limbe; 4) lived more than 150 km from Limbe; and 5) were residents of Douala (74 km away on a main road). Patient records did not provide information about missed doses of medication. Adherence was measured by the number of clinic visits attended, and defined as "good" if a patient attended 95% or more of scheduled clinic visits and "poor" if a patient attended between 80% and 94% of scheduled visits; "non-adherent" was defined as attending less than 80% of scheduled visits.

A patient was classified as dead if they had died on the hospital premises, or if the death had been confirmed by hospital staff or a relative. Patients were classified as lost to follow up if they failed to return for their last scheduled clinic visit and had not been seen within 91 days prior to the end date of 31 December 2005. Patients who had contact with the healthcare system, either during an initial visit or follow-up visit during the 91 days prior to 31 December 2005, were considered to be active and in care. We defined "active" patients to be those who were still recorded in the programme as of 31 December 2005, although the patient may have interrupted therapy at some time.

### Data analysis

We retrospectively analyzed clinical and pharmacy payment records of HIV-infected patients who were newly enrolled in the Limbe ART programme between February 2002 and December 2005. We stratified the overall sample into two cohorts: patients who initiated ART before 1 October 2004; and patients who initiated ART on or after 1 October 2004. For the sample overall and for each cohort, we computed medians and interquartile ranges or ranges for continuous variables and frequencies for categorical variables. Enrolments per month were compared before and after the ART price reduction using the Wilcoxon rank sum test.

We estimated unadjusted and adjusted odds ratios (ORs) using logistic regression for the association of patient characteristics with: 1) non-retention in care (dropout or death); and 2) abandoning care (dropout) immediately after initiation of ART. Separate logistic models were fit for each characteristic of interest, and then all characteristics were entered simultaneously in a multiple logistic regression model, in which ORs were adjusted for all of the other variables in the model. Wald chi-square tests were used to test for significant associations between patient characteristics and dropout, and 95% confidence intervals (CIs) were computed using the Wald method. SAS PROC LOGISTIC was used for all logistic regression analyses.

To examine the association of factors of interest with time to dropout or death, we fit Cox proportional hazards models, and estimated unadjusted and adjusted hazard ratios (HRs) and 95% CIs. Ties among time to dropout or death were handled using the exact method. SAS PROC PHREG was used to fit proportional hazards models. Using SAS PROC LIFETEST, we computed Kaplan-Meier (product-limit) curves to estimate the survivor function for remaining alive and in care.

For 25% of the patients, at least one of the covariates of interest was missing. To avoid potential bias that may have resulted due to the exclusion of missing data, we used multiple imputation to estimate missing values. Ten imputation datasets were created using a Markov Chain Monte Carlo method with 400 burn-in iterations before the first imputation [14]. The logistic regression and survival analysis models were repeated using the imputed data, and parameter estimates for the 10 imputed datasets were combined using Rubin's methods [15]. All analyses were completed using SAS 9.1.2, (2004, SAS Institute Incorporated, Cary, NC, USA).

Ethical and administrative approvals for this study were respectively obtained from the Cameroon National Ethics Committee and the Division of Operational Research of the Cameroon Ministry of Public Health. Following these approvals, the project was determined to be non-research by the National Center for HIV, Viral Hepatitis, STD, and TB Prevention, Centers for Disease Control and Prevention, Atlanta, Georgia, USA. The study consisted of a retrospective analysis of clinical and pharmacy payment records of HIV-infected patients and therefore did not involve any consent procedure.

## Results

### Patients' baseline characteristics upon enrolment in ART

Between February 2002 and December 2005, 2920 patients (62% females) initiated ART in the Limbe ART programme (median follow-up time 6.2 months) (Table 1), contributing a total of 2463 person-years of follow up: 1926 person-years for patients enrolled before the ART price reduction on 1 October 2004 and 537 person-years for patients enrolled after the ART price reduction. Records of some variables of interest, including patients' age (4%), occupation (6%), marital status (5%), residence distance from ART clinic (4%), means to pay for antiretroviral drugs (20%) and entry CD4 cell count (7%), were missing (Tables 1, 2, 3 and 4).

**Table 1:** Demographic characteristics of patients initiating treatment in the Limbe ART programme, 2002 to 2005

	All patients [n(%), N = 2920]	Patients enrolled before 1 Oct 2004 [n(%), N = 1487]	Patients enrolled after 1 Oct 2004 [n(%), N = 1433]
Age category*			
< 30**	723 (25.8)	357 (26.0)	366 (25.6)
30+	2081 (74.2)	1017 (74.0)	1064 (74.4)
**Median age, years (range)**	35 (0.5-73)	35 (1-73)	35 (0.5-73)
Female	33 (0.7-73)	34 (1-71)	33 (0.7-72)
Male	38 (0.5-73)	38 (1-73)	37 (0.5-73)
Sex*			
Female	1815 (62.3)	886 (59.6)	929 (64.8)
Male	1104 (37.7)	600 (40.4)	504 (35.2)
**Median follow-up time, months (range)**	6.2 (0.03-46.3)	15.8 (0.03-46.3)	3.4 (0.03-14.5)
**Person-years follow up**	2462.5	1925.6	536.9
**Patients receiving treatment**			
ART naïve	2864 (98.1)	1458 (98.0)	1406 (98.1)
Patients previously on ART who were transferred from other centres	56 (1.9)	29 (2.0)	27 (1.9)
Occupation*			
Gainfully employed	2044 (74.3)	969 (73.2)	1075 (75.3)
Unemployed/dependent	706 (25.7)	354 (26.8)	352 (24.7)
Marital status*			
Married	1166 (42.0)	577 (42.9)	589 (41.1)
Single	1207 (43.5)	570 (42.4)	637 (44.5)
Previous marriage	404 (14.5)	197 (14.7)	207 (14.5)
Residence by distance to ART clinic*			
Residents in Limbe town	621 (22.1)	311 (22.7)	310 (21.7)
Residents < 40 km from Limbe	801 (28.6)	395 (28.8)	406 (28.4)
Residents 40-150 km from Limbe	788 (28.1)	384 (28.0)	404 (28.2)
Residents > 150 km from Limbe	454 (16.2)	217 (15.8)	237 (16.5)
Residents from Douala, 74 km away***	139 (5.0)	64 (4.7)	75 (5.2)
Means of payment for antiretroviral drugs*			
Entirely paid by programme or employer	87 (3.7)	48 (4.1)	39 (3.4)
Paid by self, family or partly by other	2241 (96.3)	1134 (95.9)	1107 (96.6)

*Sample size smaller due to missing values

** 26 clients were < 5 years old; 20 clients were 6-10 years old; 47 clients were 11-19 years old

*** Patients residing in Douala with regular transport on paved roads and may have had other ART opportunities Abbreviations: ART (antiretroviral therapy)

**Table 2:** Clinical status of patients enrolled in the Limbe ART programme, 2002 to 2005

	All patients [n(%), N = 2920]	Patients enrolled before 1 Oct 2004 [n(%), N = 1487]	Patients enrolled after 1 Oct 2004 [n(%), N = 1433]
**Final clinical status (as of 31 December 2005)**			
In care	1597 (54.7)	663 (44.6)	934 (65.2)
Lost to follow up	1149 (39.3)	715 (48.1)	434 (30.3)
Dead	164 (5.6)	104 (7.0)	60(4.2)
Transferred	10 (0.3)	5 (0.3)	5 (0.3)
**Adhering to ART clinic visits**			
< 80% (non-adherence)	2260 (77.4)	1286 (86.5)	974 (68.0)
80-94% (poor adherence)	365 (12.5)	158 (10.6)	207 (14.4)
> 94% (good adherence)	295 (10.1)	43 (2.9)	252 (17.6)
**Dropped out of care immediately after ART initiation**			
Yes	631 (21.6)	295 (19.8)	336 (23.5)
No	2289 (78.4)	1192 (80.2)	1097 (76.5)
**Tuberculosis co-infection at the beginning or during the course of ART**			
Yes	35 (1.2)	20 (1.3)	15 (1.01)
No	2885 (98.8)	1467 (98.6)	1418 (98.9)
**Median entry CD4 count, cells/mm^3 ^(range)**	107 (0-1696)	101 (0-1696)	111 (0-958)
Entry CD4 count, (cells/mm^3^)*			
0-99	1287 (47.4)	659 (49.1)	628 (45.7)
> 100	1428 (52.6)	682 (50.9)	746 (54.3)
Treatment regimen on enrolment*			
Stavudine/lamivudine/nevirapine	2752 (94.7)	1390 (94.3)	1362 (95.0)
Stavudine/lamivudine/efavirenz	40 (1.4)	32 (2.2)	8 (0.6)
Zidovudine/lamivudine/nevirapine	25 (0.9)	4 (0.3)	21 (1.5)
Zidovudine/lamivudine/efavirenz	82 (2.8)	40 (2.7)	42 (2.9)
Stavudine/lamivudine/indinavir	5 (0.2)	5 (0.3)	0
Zidovudine/lamivudine/indinavir	3 (0.1)	3 (0.2)	0

*Sample size smaller due to missing values

Abbreviations: ART (antiretroviral therapy)

**Table 3:** Factors associated with time to dropout or death among patients enrolled in the Limbe ART programme, Feb 2002-Dec 2005

		Complete Cases				Multiple Imputation	
**Characteristic**	**n/N**	**%**	**Crude HR**	**Adjusted HR**	**Missing N(%)**	**Crude HR**	**Adjusted HR**
			**(95% CI)**	**(95% CI)**		**(95% CI)**	**(95% CI)**

**Age group**							
30+	880/2081	42.3	1.00	1.00	116	1.00	1.00
< 30	350/723	48.4	1.19 (1.03, 1.37)	1.20 (1.03, 1.43)	(3.97)	1.22 (1.08, 1.34)	1.25 (1.10, 1.45)
Sex*							
Female	760/1815	41.9	1.00	1.00	1	1.00	1.00
Male	552/1104	50.0	1.20 (1.06, 1.36)	1.31 (1.14, 1.50)	(0.03)	1.20 (1.07, 1.34)	1.33 (1.18, 1.50)
Occupation*	888/2044	43.4	1.00	1.00	170	1.00	1.00
With some kind of income							
Unemployed	316/706	44.8	1.04 (0.90, 1.20)	1.06 (0.91, 1.23)	(5.82)	1.01 (0.89, 1.15)	1.03 (0.90, 1.19)
Marital status*							
Married/living as	501/1166	43.0	1.00	1.00	143	1.00	1.00
Never	558/1207	46.2	1.09 (0.96, 1.25)	1.10 (0.95, 1.27)	(4.90)	1.14 (1.01, 1.28)	1.11 (0.98, 1.27)
Previous marriage	158/404	39.1	0.85 (0.70, 1.04)	0.94 (0.76, 1.16)		0.91 (0.76, 1.09)	1.01 (0.84, 1.21)
Residence by distance to ART clinic*							
Residents in Limbe	239/621	38.5	1.00	1.00	117	1.00	1.00
Residents < 40 km from Limbe	339/801	42.3	1.13 (0.94, 1.37)	1.08 (0.89, 1.31)	(4.01)	1.13 (0.96, 1.33)	1.05 (0.89, 1.23)
Residents 40-150 km from Limbe	349/788	44.3	1.15 (0.95, 1.39)	1.13 (0.93, 1.36)		1.18 (1.00, 1.39)	1.12 (0.95, 1.32)
Residents > 150 km from Limbe	236/454	52.0	1.63 (1.33, 2.00)	1.57 (1.28, 1.93)		1.52 (1.27, 2.51)	1.41 (1.18, 1.69)
Residents from Douala, 74 km away***	72/139	51.8	1.79 (1.33, 2.40)	1.71 (1.27, 2.30)		1.66 (1.28, 2.15)	1.51 (1.16, 1.98)
Means of payment for antiretroviral drugs*							
Entirely paid by programme or employer	15/87	17.2	1.00	1.00	592	1.00	1.00
Paid by self, family or partly by other	1036/2241	46.2	3.12 (1.67, 5.82)	3.34 (1.79, 6.25)	(20.27)	2.95 (1.80, 4.85)	3.05 (1.99, 4.67)
**Cohort**							
Enrolled after 1 Oct 2004	494/1433	34.5	1.00	1.00	0	N/A	1.00
Enrolled before 1 Oct 2004	819/1487	55.1	1.04 (0.91, 1.19)	1.06 (0.92, 1.21)	(0.0)		1.08 (0.96, 1.22)
Entry CD4 count (cells/mm^3^)*							
> 100	543/1428	38.0	1.00	1.00	205	1.00	1.00
0-99	644/1287	50.0	1.43 (1.27, 1.61)	1.41 (1.25, 1.61)	(7.02)	1.43 (1.28, 1.61)	1.41 (1.27, 1.59)

(complete case N = 2188; multiple imputation N = 2920).

* Sample size smaller due to missing values

** Difficult access took into account the distance from ART centre as well as the presence of unpaved or poorly maintained road

*** Patients residing in Douala with regular transport on paved roads and may have had other ART opportunities

Abbreviations: ART (antiretroviral therapy)

**Table 4:** Factors associated with dropping out of care immediately after initiating ART in the Limbe ART programme, Feb 2002-Dec 2005

		Complete Cases				Multiple Imputation	
**Characteristic**	**n/N**	**%**	**Crude HR**	**Adjusted HR**	**Missing N(%)**	**Crude HR**	**Adjusted HR**
			**(95% CI)**	**(95% CI)**		**(95% CI)**	**(95% CI)**

**Age group**							
30+	441/2081	21.2	1.00	1.00	116	1.00	1.00
< 30	159/723	22.0	1.06 (0.83, 1.33)	1.07 (0.82, 1.40)	(3.97)	1.03 (0.84, 1.27)	1.09 (0.86, 1.37)
Sex*							
Male	215/1104	19.5	1.00	1.00	1	1.00	1.00
Female	416/1815	22.9	1.20 (0.77, 1.49)	1.24 (0.98, 1.57)	(0.03)	1.23 (1.02, 1.49)	1.22 (1.00, 1.50)
Occupation*							
Unemployed	137/706	19.4	1.00	1.00	170	1.00	1.00
With some kind of income	453/2044	22.2	1.12 (0.88, 1.43)	1.23 (0.95, 1.60)	(5.82)	1.19 (0.96, 1. 49)	1.27 (1.00, 1.61)
Marital status*							
Married/ living as	232/1166	19.9	1.00	1.00	143	1.00	1.00
Never	266/1207	22.0	1.07 (0.86, 1.35)	1.01 (0.79, 1.28)	(4.90)	1.14 (0.94, 1.39)	1.03 (0.84, 1.27)
Previous marriage	97/404	24.0	1.13 (0.82, 1.54)	1.05 (0.76, 1.46)		1.28 (0.97, 1.68)	1.16 (0.87, 1.54)
Residence by distance to ART clinic*							
Residents in Limbe	138/621	22.2	1.00	1.00	117	1.00	1.00
Residents < 40 km from Limbe	177/801	22.1	1.16 (0.87, 1.55)	1.16 (0.86, 1.57)	(4.01)	1.01 (0.79, 1.29)	0.95 (0.74, 1.22)
Residents 40-150 km from Limbe	156/788	19.8	0.84 (0.62, 1.15)	0.82 (0.60, 1.13)		0.87 (0.68, 1.13)	0.83 (0.64, 1.08)
Residents > 150 km from Limbe	97/454	21.4	1.06 (0.75, 1.50)	1.04 (0.74, 1.48)		0.95 (0.71, 1.28)	0.92 (0.69, 1.23)
Residents from Douala, 74 km away***	31/139	22.3	0.97 (0.57, 1.64)	1.00 (0.59, 1.69)		1.01 (0.65, 1.56)	0.95 (0.61, 1.47)
Means of payment for antiretroviral drugs*							
Entirely paid by programme or employer	14/87	16.1	1.00	1.00	592	1.00	1.00
Paid by self, family or partly by other	456/2241	20.4	0.86 (0.46, 1.61)	0.80 (0.42, 1.52)	(20.27)	1.33 (0.74, 2.39)	1.25 (0.67, 2.32)
**Cohort**							
Enrolled before Oct 1,2004	295/1487	19.8	1.00	1.00	0	N/A	1.00
Enrolled on or after Oct 1,2004	336/1433	23.5	1.06 (0.86, 1.30)	1.05 (0.85, 1.30)	(0.0)		1.22 (1.03, 1.46)
**Entry CD4 count (cells/μL)**							
> 100	296/1428	20.7	1.00	1.00	205	1.00	1.00
0-99	292/1287	22.7	1.09 (0.88, 1.34)	1.08 (0.87, 1.33)	(7.02)	1.13 (0.94, 1.35)	1.14 (0.95, 1.36)

(Complete case N = 2188; Multiple Imputation N = 2920).

*Sample size smaller due to missing values, **Difficult access took into account the distance from ART centre as well as the presence of unpaved or poorly maintained road, ***Patients residing in Douala with regular transport on paved roads and may have had other ART opportunities. Abbreviations: ART (antiretroviral therapy)

The median age of patients was 35 years (range 0.5-73 years). Most patients (74%) were employed (self or casual employment, salaried workers and farmers); the remaining patients (26%) were either unemployed or dependent on others for support. The vast majority of patients (96%) paid for ART through family and/or self support; 87 (4%) received ART free of charge either through the Global Fund programme for indigents or from their employers.

**Figure 1:**
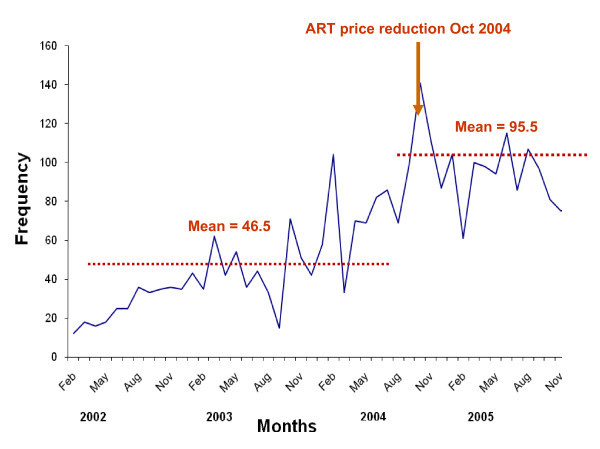
**Retention in an antiretroviral therapy programme during an era of decreasing drug cost in Limbe, Cameroon**. Monthly enrolment of patients initiating antiretroviral therapy (ART) in the Limbe ART programme, 2002-2005, Cameroon.

The monthly rate of enrolment in the Limbe ART programme generally increased over time (Figure 1) and this increase was more marked for women than for men (data not shown). The rate of enrolment was higher after the ART drug price reduction on 1 October 2004: 95.5 persons/month after the price reduction compared with 46.5 persons/month prior to the reduction (p < 0.001).

### Clinical outcomes

As of 31 December 2005, 1597 (55%) patients remained in care, 1149 (39%) were lost to follow up, 164 (6%) were documented as having died, and 10 (0.3%) had transferred care to another treatment centre (Table 2). Adherence to clinical appointments was generally poor: 77% of patients were non-adherent (i.e., attended < 80% of scheduled clinic visits); adherence for only 10% was characterized as good (attended ≥95% of scheduled visits). Notably, 22% of patients dropped out of care after enrolling and receiving their first monthly dispensation of ART.

**Figure 2:**
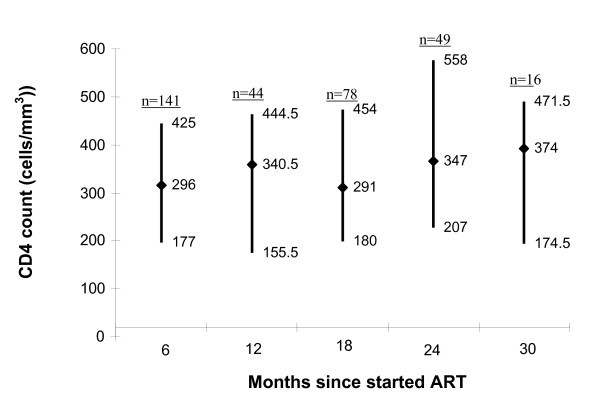
**Median (interquartile range) CD4 cell counts for patients enrolled in the Limbe antiretroviral (ART) programme measured at six-month intervals after staring ART, 2002-2005, Cameroon**. CD4 cell counts were paid for by the client and were available for relatively few persons while on ART.

Most patients initiating treatment had advanced disease, either WHO stage 3 and 4 (75%) or CDC stage B3 and C3 (86%). Although 93% of patients had a CD4 cell count measured at enrolment, only 6% had any follow-up counts measured. The median baseline enrolment CD4 cell count was 107 cells/mm^3 ^(IQR 36-181 cells/mm^3^, n = 2715). Median CD4 cell counts at six-monthly visits after baseline were > 200 cells/mm^3 ^for most patients for whom data were available (Figure 2).

First-and second-line ART regimens were prescribed following national guidelines and included nucleoside and non-nucleoside reverse transcriptase inhibitor-based and protease inhibitor-based ART. Most patients received first-line treatment regimens, which in 95% of cases was a generic fixed-dosed combination of stavudine, lamivudine and nevirapine (Triomune^®^, Cipla, Mumbai, India). Less than 0.5% of patients received any second-line regimen.

### Effect of drug price reduction on enrolment, retention in care and survival

Patients enrolled before or after the ART drug price reduction did not differ with regard to median age, sex, occupation, marital status, access to ART clinic and means to pay for drugs (Table 1). Likewise, patients enrolled before or after the ART drug price reduction did not differ with regard to dropping out of care after initiating ART, tuberculosis co-infection at the beginning or during the course of therapy, baseline CD4 cell count and treatment regimen received (Table 2). Adhering to ART clinic visits was good for few patients enrolled both before (2.9%) and after (17.6%) the price reduction.

**Figure 3:**
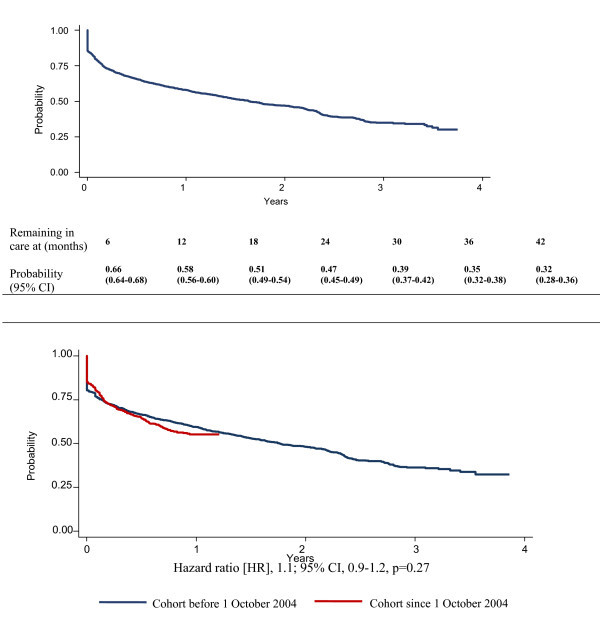
**Figure 3a: Kaplan-Meier survival estimates of remaining alive and in care of patients initiating antiretroviral therapy (ART) in the Limbe ART programme, February 2002-December 2005**. Figure 3b: Kaplan-Meier survival estimates of remaining alive and in care in the Limbe antiretroviral therapy (ART) programme, 2002-2005, by two cohorts distinguished by a major drug price reduction on 1 October 2004.

The probabilities of remaining alive and in care were 0.66 (95% CI 0.64-0.68) at six months, 0.58 (95% CI 0.56-0.60) at one year, 0.47 (95% CI 0.45-0.49) at two years and 0.35 (95% CI 0.32-0.38) at three years (Figure 3a). These probabilities were not significantly different when comparing patients enrolled before with patients enrolled after the drug price reduction over the first 15 months of comparable follow up (HR 1.1; 95% CI 0.9-1.2) (Figure 3b).

In multivariable analysis using multiple imputations to compensate for missing values, compared with residents of Limbe, those living more than 150 km from Limbe (HR 1.41 [1.18-1.69], p < 0.001) or those who were residents of Douala (HR 1.51 [1.16-1.98], p < 0.001) were more likely to be lost to follow up or die (Table 3). Patients who paid for care themselves or for whom care was paid by their family or others were more likely to be lost to follow up or die compared with patients whose care was paid entirely by an employer or an ART programme, such as the Global Fund (HR 3.05; 95% CI 1.99-4.67). Men were more likely to drop out of care or die than women (HR 1.33; 95% CI 1.18-1.50). Other factors associated with dropping out of care or dying in multivariable analysis included age less than 30 years (HR 1.25; 95% CI 1.10-1.45) and entry CD4 cell count of less than 100 cells/mm^3 ^(HR 1.41; 95% CI 1.27-1.59).

Patients who were lost to follow up after the initial visit were more likely to be female (OR 1.22; 95% CI 1.00-1.50), to have been enrolled on or after the antiretroviral drug price reduction on 1 October 2004 (OR 1.22; 95% CI 1.02-1.46), and to have been gainfully employed (OR 1.27; 95% CI 1.00-1.61) (Table 4).

## Discussion

A major reduction in the price of antiretrovirals in Cameroon was associated with an increased number of HIV-infected patients initiating ART, but did not appear to be associated with retention in care in this setting where most patients had to pay for their own treatment. Retention in the Limbe ART programme decreased substantially over time: less than 50% of patients remained in care at two years and only one-third at three years. The proportions of patients remaining in care did not differ according to when treatment was started (i.e., before or after 1 October 2004).

Though we cannot prove that the reduction in price of ART was the only variable associated with the outcome, the socio-economic conditions of the country did not change appreciably during the period of the study. Paying for ART in our study constituted a major increase in the risk of dropout or death, and our findings are consistent with other studies, which have demonstrated that providing ART in resource-constrained settings where people have to pay for their own treatment was associated with high dropout rates among persons with advanced baseline disease [16,17]. Other studies have found self-paid programmes were also associated with failure to achieve viral suppression [18]; however, we were unable to examine this outcome because viral load testing was not available in Southwest Cameroon at that time.

Affordability of antiretroviral treatment is not the only factor that can influence retention or adherence to treatment. For example, laboratory monitoring required for patients on ART at that time cost US$30 in Cameroon and was not readily affordable. The majority of patients had their CD4 cell counts measured as a requirement for entry into the treatment programme, but very few paid for follow-up CD4 cell count testing. For the few patients who paid for follow-up CD4 cell counts, the increases we observed during follow up were consistent with improved clinical outcome. Most patients presented for care with late-stage disease, possibly due to the expense of treatment and insufficient access to voluntary counselling and testing services. The late entry to care we observed in the present study is consistent with the findings of another Cameroonian report that found patients usually first learn their HIV status late in the course of infection when they have already developed clinical signs and symptoms of AIDS [19].

Poor adherence is associated with an increased risk of mortality [20]. Good adherence, retention in care and survival have been achieved through such interventions as home-based AIDS care [21-23] and participant-identified support partners [24]. Adherence in the present study, measured as attendance to scheduled clinic visits, was generally poor, with only 10% of patients attending 95% or more of their visits. More than 20% of patients were lost to follow up after their initial ART visit, which was due, in part, to early deaths. The magnitude of this effect (i.e., early death after ART initiation) was difficult to quantify since for some patients, the date of death was unknown, and some patients who were classified as lost to follow up may have died.

Expanded access to antiretroviral therapy is feasible in urban sub-Saharan Africa [24]. Increased distance from the Limbe ART clinic was associated with poor retention in care. This has also been demonstrated by others in the United States [25]. Patients who traveled to Limbe from Douala, which had at least two ART programmes at that time, were at greater risk of becoming lost to follow up. This might have resulted from differences in the antiretroviral treatment options and the quality of care that were available in Limbe compared with Douala. Regardless, expanding antiretroviral treatment programmes to be closer to more people can overcome the obstacle of distance from clinics that may impede attendance.

Few patients had their antiretroviral drug costs and laboratory tests paid entirely by their employer or an ART programme. Most of these persons were employees of a local oil refining company located in Limbe, and had good access to the clinic.

A reduction in antiretroviral drug price in our study was associated with increasing numbers of patients accessing ART, but was not associated with improved retention in care. However, free treatment, which included payment for laboratory monitoring, was associated with improved retention in care. This further validates the approach of the current era of antiretroviral treatment programmes in Africa that are largely donor supported and provide treatment and care to persons living with HIV disease for minimal or no charge. Requiring persons with limited resources to pay for such care would not have been successful or sustainable in the long term.

A strength of our analysis is its large size; we assessed data from a large ART programme with good record keeping and a considerable length of follow-up time for patients, and we had access to patients' pharmacy and clinical payment records. However, our analysis is subject to some limitations. Data were collected retrospectively and there was a low rate of immunologic follow up since clients had to pay for the test and most could simply not afford it; thus the CD4 cell counts available may not reflect the CD4 cell counts of all clients while on ART.

The extremely high rate of loss to follow up, particularly after only one visit, where the outcomes of these patients are not known, may have contributed to underestimation of mortality in particular. A follow-up tracing study to characterize patients receiving ART who were lost to follow up may be useful to enable us to understand the large proportion of those who were lost only after one visit. There was no virologic monitoring available during that time in this part of Cameroon. To overcome potential bias resulting from exclusion of missing data, we employed multiple imputation to generate estimated values for missing data and the abandoned care analyses found that cohort was a predictive factor only when imputed data was included.

## Conclusions

We found that proximity to care was associated with improved outcomes, regardless of the cost of therapy. However, many patients sought care late in the course of their infection, did not return for scheduled clinic visits, and were lost to care. If poor retention in care is a proxy for poor adherence to taking antiretroviral drugs as prescribed, then our findings should raise concern that in programmes where patients must pay for their own care, selective pressure favouring drug resistance might be increased.

Cameroon now has an ongoing policy to expand free ART that should improve survival and retention in care. Decentralizing ART clinics and linking them with increased voluntary counselling and testing services may reduce late presentation to care and thereby further improve survival and retention in care in Cameroon. The data from this report will serve as an historical comparison to the current approach that provides free ART.

## Competing interests

The authors declare that they have no competing interests.

## Authors' contributions

JJM conceived and designed the study, drafted the manuscript, and participated in data analysis and interpretation. WA participated in the design of the study, performed acquisition of data, and helped to draft the manuscript. PJW helped to conceive and design the study, helped to draft the manuscript, revising it critically for important intellectual content, and participated in statistical analysis and interpretation. JTB helped to draft the manuscript, revising it critically for important intellectual content, and participated in statistical analysis and interpretation. AJA helped in acquisition of data, participated in critical data organization, cleaning and preliminary analysis, and helped to draft the manuscript. TNK helped to conceive and design the study, and to draft the manuscript, revising it critically for important intellectual content. SP participated in drafting the manuscript and performed statistical analysis. PLR provided significant input in conceiving and designing the study, participated in drafting the manuscript, revising it critically for important intellectual content, and participated in data analysis and interpretation. All authors read and approved the final manuscript.

## Authors' information

Jembia J Mosoko: MD, MSc; Wilfred Akam: MD; Paul J Weidle: Pharm D, MPH; John T Brooks: MD; Asabi J Aweh: MSc; Thompson N Kinge: MD; Sherri Pals: PhD; and Pratima L Raghunathan: PhD, MPH.

Jembia J Mosoko, MD, MSc, is currently affiliated with the Division of Global HIV/AIDS, Center for Global Health, CDC-Cameroon, and Pratima L Raghunathan with the Division of Global HIV/AIDS, Center for Global Health, Centers for Disease Control and Prevention, Kigali, Rwanda.
